# Electronic properties of lithium-ion battery cathodes studied in ion-gated transistor configuration

**DOI:** 10.1016/j.isci.2022.105888

**Published:** 2022-12-27

**Authors:** Federico Poli, José Ramón Herrera, Tian Lan, Prajwal Kumar, Clara Santato, Francesca Soavi

**Affiliations:** 1 Dipartimento di Chimica “Giacomo Ciamician”, Alma Mater Studiorum Università di Bologna, 40132, Bologna, Italy; 2 Engineering Physics, Polytechnique Montreal, 2500 Chemin de Polytechnique, Montreal, H3T 1J4, Canada

**Keywords:** Electrochemistry, Applied sciences, Energy storage

## Abstract

Electronic and ionic transport governs lithium-ion battery (LIB) operation. The in operando study of electronic transport in lithium-ion transition metal oxide (LMOx) cathodes at different states of charge enables the evaluation of the state of health of LIBs and the optimization of their performance. We report on electronic transport in LIB cathode materials at different states of charge controlled in operando in ion-gated transistor (IGT) configuration. We considered LiNi_0.5_Mn_0.3_Co_0.2_O_2_ (NMC532)- and LiMn_1.5_Ni_0.5_O_4_ (LNMO)-based composite materials formulated like in conventional LIB cathodes and operated in the organic electrolyte LP30 (1M LiPF_6_ in ethylene carbonate:dimethyl carbonate 1:1 v/v). NMC532- and LNMO-based cathode materials were used as the transistor channel materials and LP30 as the ion gating medium. Beyond its impact on the field of LIBs, our work advances the design of novel devices based on mixed ionic and electronic transport, including neuromorphic computing.

## Highlights

• The electronic conductivity of Lithium-ion battery electrodes is detected operando
• Lithium-ion battery cathodes are used as ion-gated transistor channel materials
• The electronic conductivity of the cathodes increases with their delithiation
• Ion-gated transistors enable evaluation of the lithium-ion battery state of health

## Graphical abstract

**Figure:**
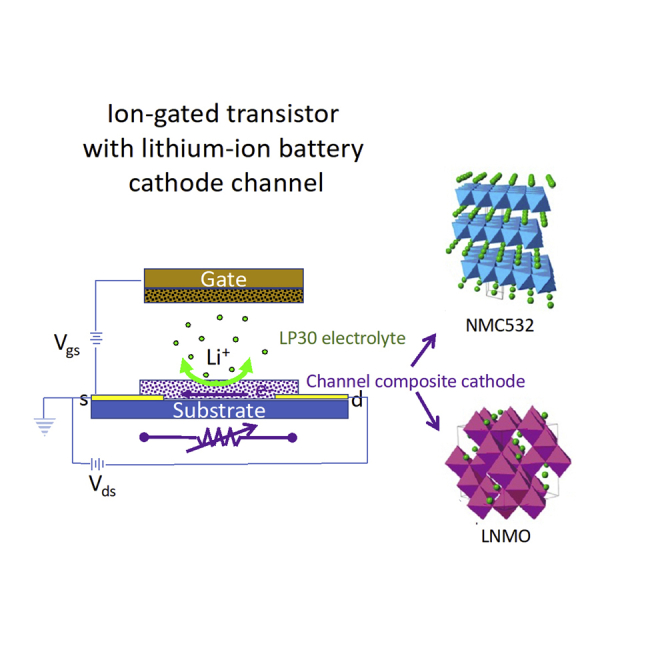

## Introduction

Lithium-ion insertion materials (LIMs) play a key role in the ongoing energy and digital industrial revolutions. These materials are core components of lithium-ion batteries (LIBs), the high-performance energy storage technology that is accelerating the path toward a zero-carbon society. LIBs are also paving the way to next generation, lowpower electronics.^1^^,^^2^

LIMs store charge by reversible host (crystal lattice)-guest (Li^+^) reactions, assumed to take place with negligible variation of crystal lattice parameters. During charge/discharge, lithium ions move in the LIM lattice whereas electrons are transferred to the external cell circuit. This requires that LIMs simultaneously exhibit electronic and Li-ion conductivity, and thus are termed mixed electronic-ionic conductors.^3^

In modern LIBs, lithium-ion transition metal oxides (LMOx, with M = Ni, Mn, Co) are used as cathode materials.^4^^,^^5^ The electronic conductivity of LMOx is important when the charge is stored/delivered at high current rates, a requirement for high-power LIBs. In addition, cycle life, aging, safety, and reliability are key features of LIBs that are affected by the evolution of the electronic properties of the LIMs with battery operation. It is worth noting that undesirable structural changes of the LIMs might affect the electrical contacts between the LIM and the current collector, bringing about an increase of LIB impedance and a decrease of cell capacity with cycling.^4^^,^^5^

During the first charge/discharge cycles (*LIB formation cycles*), a passive layer, indicated as the solid electrolyte interface (SEI), forms at the electrode/electrolyte interfaces. The Li-ion mobility within SEI and the stability of SEI are crucial for LIB operation. Although SEI is always present in LIB graphite anodes, it is also important for high voltage cathodes such as Ni-rich LMOx. These cathodes can operate above the anodic stability limit of the electrolyte, thus inducing side reactions involving the oxidative decomposition of the electrolyte; a cathode/electrolyte interface (CEI) forms that protects the cathode from further decomposition reactions and enables stable operation at high voltages.^6^ The CEI is electronically insulating and, in principle, Li-ion conductive. Therefore, it affects the cathode and overall cell impedance as well as the LIB current response, cyclability and safety. Monitoring the LIB cell internal impedance and, specifically, the evolution of the electronic and ionic conductivity of LIMs during operation, represents a diagnostic approach for the evaluation of the state of health of the batteries, which is needed to operate them properly, efficiently, and safely.^7^ Smart sensors for the evaluation of battery degradation require further developments and should be implemented in modern batteries packages to guarantee the requested robustness and reliability.^8^

The intrinsic high reversibility and fast kinetics of electron transfer in LIMs are triggering the exploitation of LIMs in an emerging area termed lithium-iontronics. Lithium-iontronic sensors, memristors, and neuromorphic devices leverage the dependency of the electronic properties of LIM electrodes on lithiation degree. As an example, Li_x_CoO_2_, Li_4_Ti_5_O_12,_ and Li_7_Ti_5_O_12_ have been investigated as functional materials for memristors.^2^^,^^9^^,^^10^ Furthermore, LIMs have been employed as components of Systems on a Chip (SoC), integrating electrical switch, energy harvesting, and storage functions.^11^

Electrochemical impedance spectroscopy (EIS) is a first choice technique to investigate ionic and electronic transport in LIBs. For a full LIB featuring a LIM cathode and a graphite anode, the Nyquist diagram describes the contribution to the full-cell impedance of different processes that take place at different time scales. At a very high frequency, the response is dominated by the ohmic resistance of the cell that comprises the electronic resistance of the electrode (whose composition includes redox active materials, carbon conductive additives, binder and current collector) and the ionic resistance of the electrolyte/separator interface. At medium frequencies, it is possible to evaluate the impedance related to SEI and CEI and the electrode charge transfer processes.^12^^,^^13^^,^^14^ Lithium-ion diffusion through the electrode active materials can be observed at very low frequencies.^15^

EIS has indicated that cell impedance evolves during LIB charge/discharge.^16^^,^^17^ However, as described above, cell impedance results from several processes that often take place on a similar timescale, and it is challenging to evaluate every single contribution without direct measurement.

To clarify the relation between LIB state of charge and impedance, *ex situ* EIS studies have been carried out on single electrodes, based on composite or pure active materials, featuring different lithiation degrees and crystal lattice parameters.^18^^,^^19^ For high-operating potential cathodes, such as Li_(1-x)_Ni_0.33_Mn_0.33_Co_0.33_O_2_ (NMC111) and Li_(1-x)_Ni_0.5_Mn_0.2_Co_0.3_O_2_ (NMC523), EIS measurements run on NMC523 and NMC111 pellets at different degree of lithiation showed that both materials exhibit semiconductor-like behavior with a thermally activated conductivity characterized by an activation energy of 0.4–0.05 eV and an electronic conductivity that increases with the de-lithiation (i.e., with the increase of the oxidation state and electrode potential versus Li^+^/Li).^18^ Starting from the fully lithiated condition, there is an increase of the electronic conductivity (ca. 10%) in the potential region where the redox Ni^4+^/Ni^3+^ process occurs, followed by a sharp conductivity increase (ca. 75%) that is related to the Co^4+^/Co^3+^ redox process. A similar approach was used to evaluate the change of the electronic properties of the spinel LiNi_x_Mn_2-x_O_4_ (LNMO) as a function of its lattice parameters which, in turn, changed with lithium-ion content. LNMO pellets were at first galvanostatically lithiated ex situ, at different lithium-ion contents, in an electrochemical cell featuring a metallic lithium counter electrode. Afterward, the electrolyte was removed from the pellets, which, after drying, were placed between two blocking electrodes for EIS studies at different temperatures.^19^ Despite these achievements, literature mainly proposes indirect methods for the evaluation of the electronic properties of LIMs, and none of them enables an in operando analysis.^18^^,^^19^ Furthermore, the studies focus on LIM powders that rarely are used alone in devices. Typically, LIMs are mixed with carbon conductive additives and polymer binders to yield composites cast on current collectors. The quality of these composites in terms of dispersion of the components, resistance at grain boundaries, and morphology affect the overall electronic and ionic impedance.^16^^,^^17^

Ion-gated transistors (IGTs) are iontronic devices making use of an ion gating medium, e.g., an ionic liquid or a saline solution, instead of conventional dielectric gating media, such as SiO_2_, typically used in field-effect transistors. In IGTs, at low gate-source potentials, V_gs_ (ca.−1 V), if no faradaic reactions take place, electrical double layers with specific capacitance (i.e., the amount of charge stored over a 1 V voltage bias) as high as 100 μF cm^−2^ are observable at the ion-gating medium/transistor channel interface. This high capacitance brings about high induced charge carrier density, as high as 10^15^ cm^−2^, possibly associated with electronic phase transitions in the channel material.^20^^,^^21^^,^^22^

Literature reports that the nature of the electrolyte, e.g., ion size and, for molecular ions, ion molecular structure, affects the electronic properties of transistor channel materials including metal oxides like WO_3_,^22^ TiO_2_^23^^,^^24^ and SnO_2_.^25^ Furthermore, the presence of lithium-ions in the ionic liquid was investigated for 1-Ethyl-3-methylimidazolium (EMIM^+^) bis(trifluoromethylsulfonyl) imide (TFSI^−^)-gated TiO_2_ transistors.^24^ The different characteristics of lithium-ions and EMIM^+^ induced two different doping mechanisms: Small lithium ions led to electrochemical doping via ion intercalation whereas large EMIM^+^ ions led to a combination of electrostatic and interface-confined electrochemical doping. The presence of lithium ions in the ion-gating medium brought about an increase in the drain-source transistor current, I_ds_, suggesting that lithiation strongly affects the electronic properties of the channel oxides. Furthermore, because of their potential to provide good switching of channel conductance by the intercalation/de-intercalation of lithium ions, Li-ion-based IGTs are emerging for analog computation.^26^

In this work, we propose to use IGTs making use of lithium intercalation materials as transistor channel materials to investigate in operando the evolution of the electronic transport properties of LIB cathode materials with their lithiation degree. As cases of study, we considered NMC532- and LNMO-based materials, featuring a typical lithium-ion battery (LIB) composite cathode composition, operated in LP30 (1M LiPF_6_ in Ethylene carbonate:dymethyil carbonate 1:1 v/v) organic electrolyte. We observed the materials by X-ray diffraction, scanning electron microscopy, and energy dispersive X-ray spectroscopy before electrochemical (cyclic voltammetry) and transistor characterizations.

## Results and discussion

### NMC532 and LNMO bulk (thick) electrodes

In our previous studies,^27^^,^^28^ we demonstrated that the lithiation/delithitation processes are highly reversible for both NMC532 and LNMO-bulk electrodes. These electrodes can be charged/discharged over hundreds of cycles without significant structural, morphological, or compositional changes, when operated in LP30 electrolyte.

**Figure 1:**
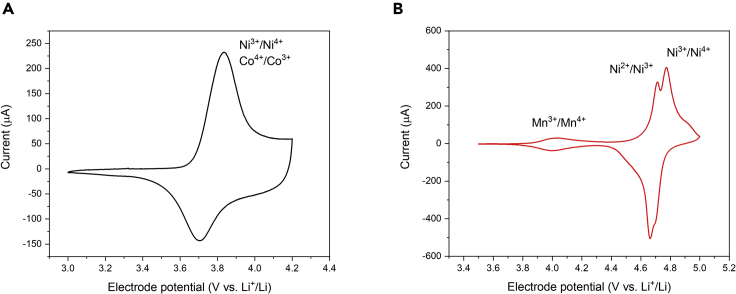
Cyclic voltammograms of bulk (A) NMC532- and (B) LNMO-based electrodes in the electrolyte LP30, in a Swagelok-like cell including a metallic lithium disk serving both counter and reference electrode. Potential scan rate 50 μV s^-1^. The redox couples associated with the observed peaks are highlighted in the voltammograms.

Figure 1 reports the cyclic voltammograms (CVs) of NMC532- and LNMO-based thick electrodes in LP30 using conventional Swagelok-type electrochemical cells. CVs were obtained after the CEI formation cycles. The CV of the NMC532 cathode (Figure 1A) shows an anodic peak located at about 3.8 V versus Li^+^/Li and a cathodic one at about 3.7 V versus Li^+^/Li. These redox peaks correspond to the Ni^4+^/Ni^3+^and Co^4+^/Co^3+^ redox couples.^27^^,^^29^ The CV of the LNMO (Figure 1B) features anodic peaks at ca. 4 V, 4.7 V, and 4.8 V versus Li^+^/Li. The corresponding cathodic peaks are located at 4 V, 4.65 V and 4.7 V versus Li^+^/Li. These reversible peaks can be assigned to the Mn^4+^/Mn^3+^, Ni^3+^/Ni^2+^, and Ni^4+^/Ni^3+^redox couples, respectively.^28^

In the case of NMC532, after the peak at 3.8 V versus Li^+^/Li, the current does not reach the baseline. Instead, it features a plateau-like behavior with a reversible (mirror-like) shape. In the potential range from 4.0 V to 4.2 V versus Li^+^/Li, the CV shows a box-shaped behavior like that of a capacitive system.^9^^,^^30^^,^^31^^,^^32^^,^^33^ This suggests that at high potentials NMC532 features a pseudocapacitive behavior attributable to the so-called *extrinsic pseudocapacitance*.^31^^,^^32^ Costentin et al. reported that the pseudocapacitive behavior can be observed when faradaic reactions, like the Li^+^ insertion/de-insertion in LMOx, bring about an evolution of the electronic structure of the material to a conductive (metallic) state, in turn leading to the formation of an electrical double layer at the electrode-electrolyte interface, as occurs in the case of capacitive electrodes.^33^ These observations agree with the conductivity change observed in de-lithiated NMC532.^18^

### NMC532-based IGTs

The structure and morphology of the NMC532 composite layer were investigated by XRD and SEM. The XRD diffraction pattern of the layer deposited on SiO_2_/Si (Figure S1) shows the single-phase layered structure expected for NMC532 (JCPDS. No. 00-85-1968).^27^ No additional reflections attributable to contaminants are observed. SEM images (Figure S2) show that NMC532, carbon particles and binder are homogenously distributed on the substrate.

**Figure 2:**
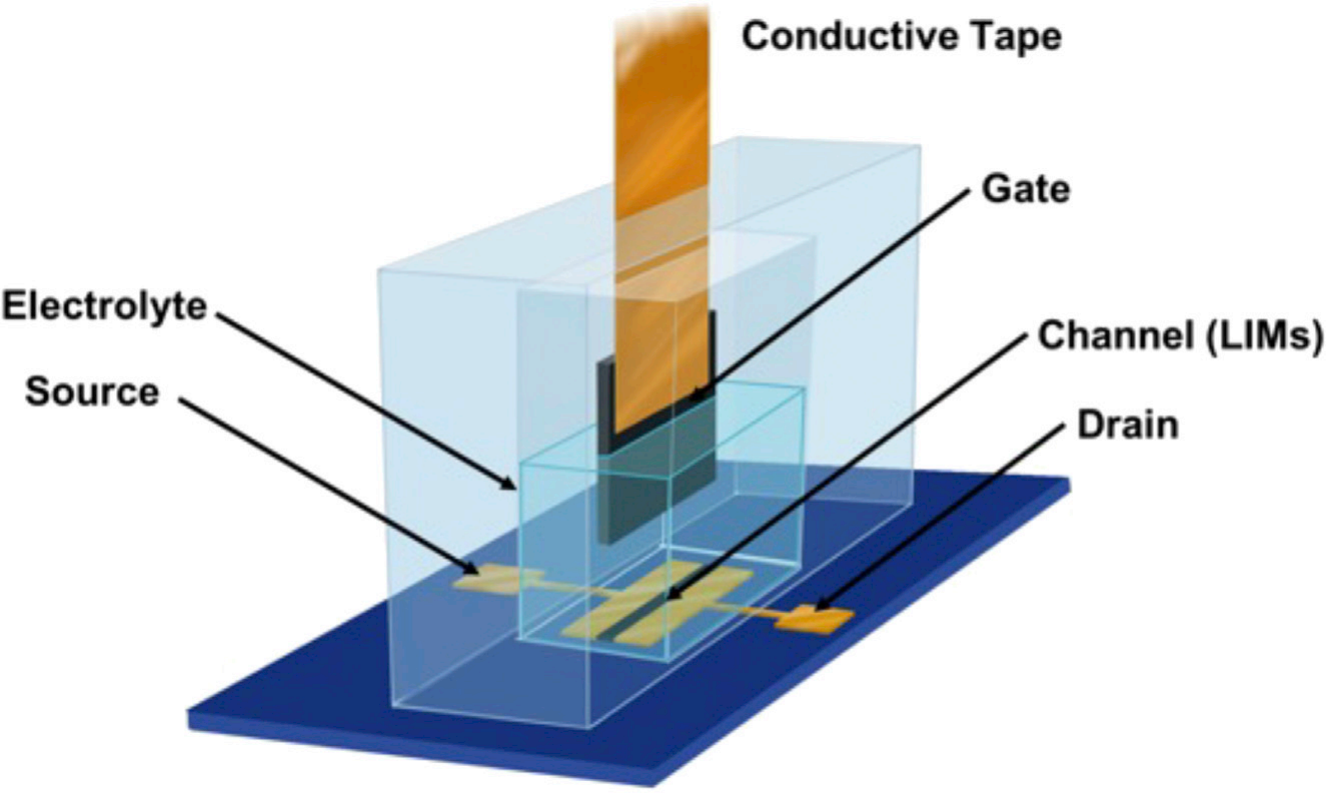
Device structure of IGTs used in this work: the transistor channel materials are lithium-ion insertion oxides (NMC532 or LNMO, LIMs), the electrolyte is LP30 (LiPF_6_ in EC:DMC), the gate is a carbon paper coated with high surface area carbon. The channel layer is deposited over the substrate (SiO_2_/Si) and between the drain and source electrodes. The IGT is housed in a PDMS frame that features an internal well filled with the electrolyte, which is in contact with channel and gate. The IGT working principle is described in Figure S5.

**Figure 3:**
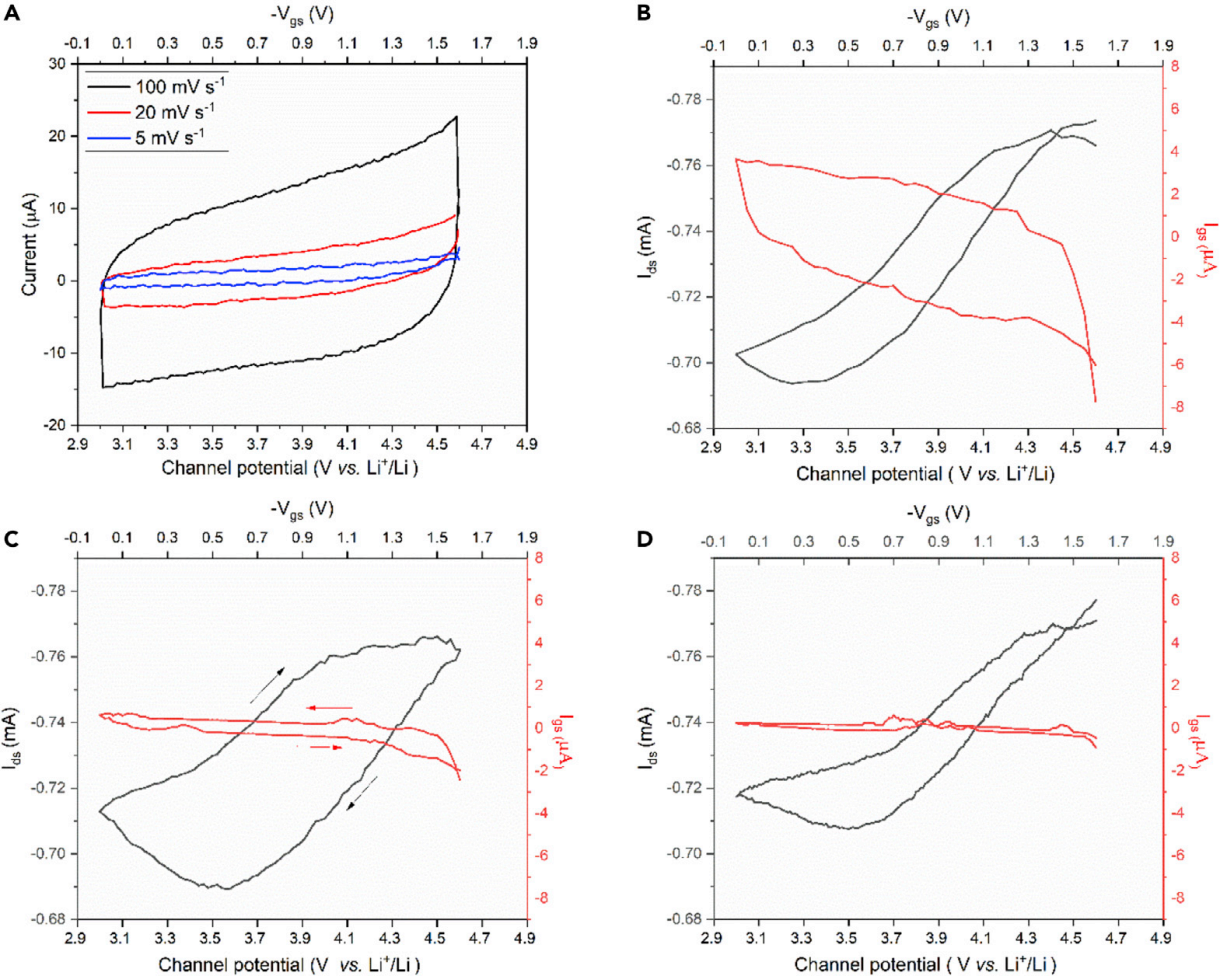
Characterization of IGTs making use of NMC532-based composite materials (see Experimental) (A) Cyclic voltammograms of the NMC532-based composite channel at different V_gs_ scan rates. Transfer curves (I_ds_ versus V_gs_, at fixed V_ds_) at V_gs_ scan rates of: (B) 100 mV s^−1^, (C) 20 mV s^−1^ and (D) 5 mV s^−1^, with V_ds_ = −200 mV.

Figure 2 describes the structure of the IGT device used to investigate in operando the electronic properties of NMC532 and LNMO. The CVs for NMC532 in IGT configuration, collected at different V_gs_ scan rates are reproted in Figure 3A. They are quasi-rectangular and quite different from the CV of the corresponding bulk electrode (Figure 1A). The voltammetric current is quasi proportional to the V_gs_ scan rate: a 5-fold increase of the current can be observed by increasing the V_gs_ scan rate from 5 mV s^−1^ to 100 mV s^−1^. Both the quasi-rectangular voltammogram shape and the linear increase of the current with the scan rate are typically observed for electrostatic charge storage at the electrical double layers. This process gives rise to a capacitive response, i.e. to the linear dependence of the charge stored within the window of potential scanned. With Faradaic processes, limited by diffusion, peak-shaped voltammograms with peak currents that increase with the square root of the scan rate, are expected. However, according to Conway,^30^ some materials, termed pseudocapacitive materials, feature the same electrochemical signature of capacitive systems, but charge storage is Faradaic, like in the case of the NMC532-IGT channel (Figure 3). Pseudocapacitance can be observed in the case of fast Faradaic reactions, not limited by solid-state diffusion processes. Hence, the NMC532-IGT voltammetric behavior can be explained by considering the thinness of the NMC532-based channel material and the evolution of the NMC532 electronic properties associated with the lithiation process. For thin transistor channels the redox reactions are surface-confined, not bulk, bringing about pseudocapacitance.^2^^,^^30^^,^^31^^,^^32^^,^^33^

Figures 3B–3D demonstrate the transfer characteristics of the NMC532-IGTs (I_ds_ versus V_gs_ for constant V_ds_); also reported is the gate-source current (I_gs_ versus V_gs_ at different V_gs_ scan rates). As already mentioned, on the one hand, I_gs_ describes a reversible electrochemical process that results from the redistribution of the ions in the electrolyte (ion gating medium) and is affected by the kinetics of the Li-ion intercalation/de-intercalation process during transistor discharging/charging.^26^ On the other hand, I_ds_ represents the current flow in the channel, from the drain to the source contact. Unlike I_gs_, I_ds_ increases above a certain V_gs_ value (the transistor threshold voltage, V_th_), with the de-lithiation (oxidation) of the NMC532-based channel. I_ds_ does not change sign during the forward and backwards sweep of V_gs._ In addition, the value of I_ds_ does not significantly depend on the scan rate, differently from I_gs_. These elements all point toward the different nature of the processes that govern I_gs_ and I_ds_. I_ds_ is not an “electrochemical current” and its value changes because the channel electronic conductivity changes on application of V_gs_, i.e., with the advancement of the de-lithiation degree (Figure S5). Such a change was previously reported only by means of ex situ analyses of bulk NMC532 pellets or EIS experiments.^18^ The values of V_th_ at the different scan rates are ca. 3.6 V versus Li^+^/Li. The ON/OFF ratio were ca. 1.1, for all the scan rates. The low values of the ON/OFF ratios can be explained considering the electrode material composition. Here, the presence of the carbon conductive additive brings about high values of I_OFF_, bringing about low ON/OFF values on application of V_gs_.

Notably, above 4.0 V versus Li^+^/Li (V_gs_ = -1 V) I_ds_ reaches a plateau. We tentatively propose that in this potential range NMC532 reaches its highest electronic conductivity state, in agreement with the voltammetric pseudocapacitive behavior of bulk NMC532 electrodes (see Figure 1A).

From the transfer curves we deduced the channel charge carrier density, n, by Equation 1.^25^

(Equation 1) $$n=\frac{Q}{e\;A}=\frac{\left(\int I_{gs}\;dV_{gs}\right)}{r_{v}\;e\;A}$$

where Q is the charge accumulated during the forward scan in the transfer curve (obtained through the integration of the gate-source current, I_gs_, versus time), A is the geometric area of the NMC532 film exposed to the electrolyte (9 ×10^−2^ cm^2^), $r_{v}$ is the V_gs_ scan rate and *e* is the elementary charge. The values of Q were 2.5 × 10^−5^ C (5 mV s^−1^), 3.4 × 10^−5^ C (20 mV s^−1^) and 4.1 × 10^−5^ C (100 mV s^−1^). The charge carrier densities we obtained at scan rates of 5, 20 and 100 mV s^−1^ were *ca.* 1.7 × 10^15^, 2.3 × 10^15^ and 2.8 × 10^15^ cm^−2^. The charge carrier mobility, *μ*, was obtained through $\mu =\frac{L}{W}\frac{I_{ds}}{{n\;e\;V}_{ds}}$ where *L* is the source and drain interelectrode distance, 10 μm, and *W* is the width, 4 mm. The values of the mobility for NMC532-based transistor channel material at scan rates of 5, 20 and 100 mV s^−1^ were ca. 3.4 × 10^−2^, 2.5 × 10^−2^ and 2.1 × 10^−2^ cm^2^ V^−1^ s^−1^.

The highest charge carrier density was about 2.8 × 10^15^ cm^−2^. It is worth noting that this value refers to the NMC532-carbon-PVdF composite layer. Being affected by the presence of the binder and the carbon additive, the charge carrier density value cannot be attributed to the NMC532 active powder alone.

NMC532-based IGTs were further studied for their output characteristics between 0 and -1.6 V, corresponding to channel potentials ranging from 3 V versus Li^+^/Li to 4.6 V versus Li^+^/Li (Figure S6). In this potential range, the reversible de-lithiation/lithiation of NMC532 takes place (Figure 1A). The linear response of I_ds_ with V_ds_ indicates the ohmic nature of the NMC532-based composite channel material, which includes the conductive carbon additive. The resistance of the channel (R_ds_) can be calculated by the slope of the I_ds_ curves as a function of V_ds_, being $\Delta V_{ds}=R_{ds}\cdot \Delta I_{ds}$ (Table S1). $R_{ds}$ decreases by bringing the channel toward more positive values versus Li^+^/Li, i.e. with the decrease of the degree of lithiation of the NMC532-based channel (Table S1). This finding agrees with other works reported in the literature that, however, have been obtained by conventional *ex situ* or EIS techniques.^18^

### LNMO-based IGTs

The XRD pattern of the LNMO composite deposited on the IGT SiO_2_/Si substrate (Figure S3) corresponds to the Fd3m cubic spinel structure (JCPDS No. 32-0581), expected for LiNi_0.5_Mn_1.5_O_4_.^28^ SEM images (Figure S4) indicates that LNMO particles are well dispersed in the carbon-binder matrix and the composite layer is homogenously distributed on the substrate.

LNMO-based cathodes feature reversible de-lithiation up to 5 V versus Li^+^/Li (see Figure 1B). At these high potentials, typically, side reactions involving electrolyte oxidative decomposition occur. Such reactions lead to the formation of a CEI^5^ possibly affecting the electronic response of the LNMO channel, therefore the upper transistor channel potential was kept lower than 4.6 V versus Li^+^/Li. The transfer curves of LNMO IGTs were collected by sweeping V_gs_ from 0.3 to −1.4 V, which corresponds to channel potentials ranging from 2.7 V to 4.4 V versus Li^+^/Li. In this potential range, only the Mn^4+^/Mn^3+^ redox process occurs.

**Figure 4:**
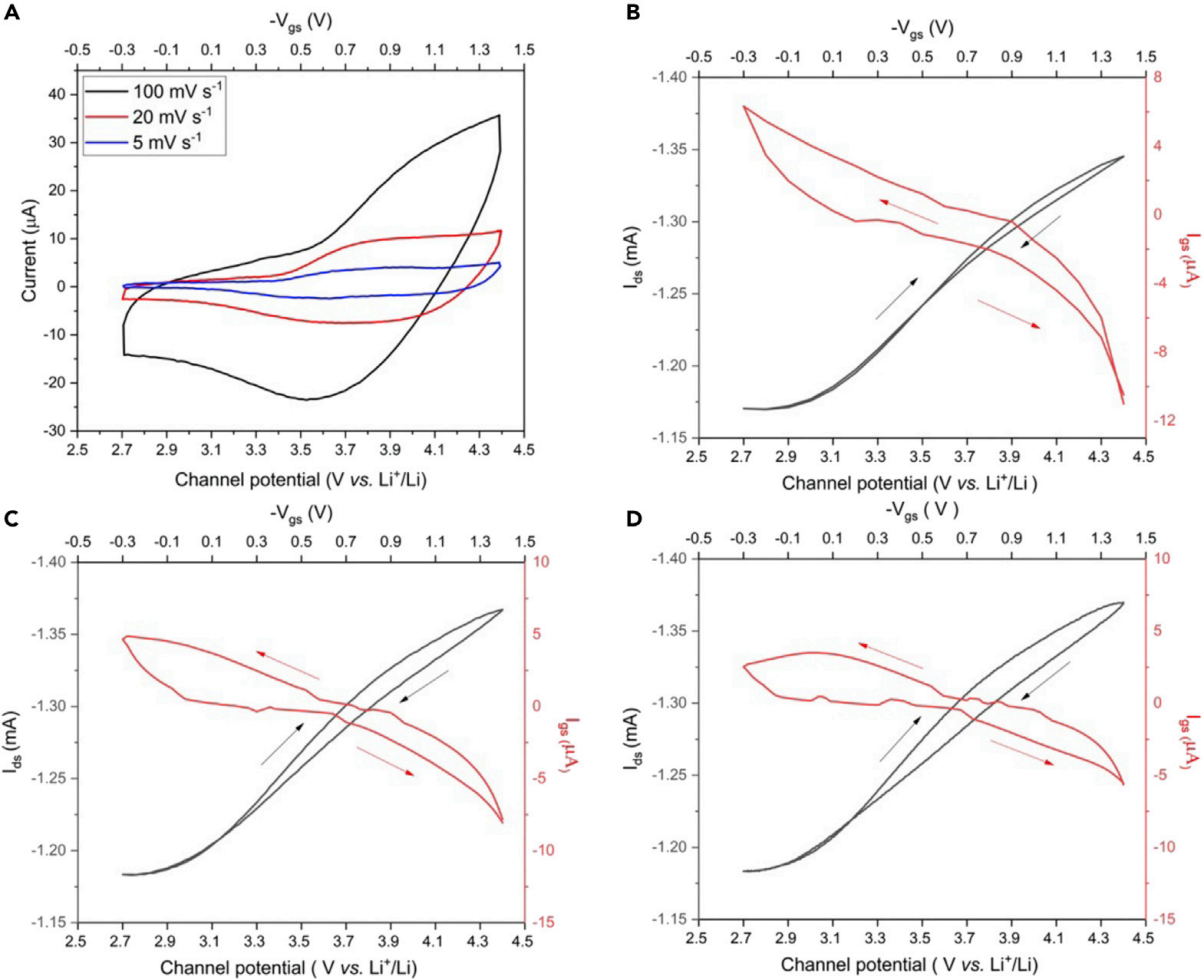
Characterization of IGTs making use of LNMO-based composite material (see Experimental) (A) Cyclic voltammograms of the LNMO-based composite channel at different V_gs_ scan rates. Transfer curves (I_ds_ versus V_gs_, at fixed V_ds_) at V_gs_ scan rates of: (B) 100 mV s^−1^, (C) 20 mV s^−1^ and (D) 5 mV s^−1^, with V_ds_ = −200 mV.

The CVs of the LNMO channel at different V_gs_ scan rates exhibit broad anodic and cathodic peaks located between 3.5 and 4.3 V versus Li^+^/Li, ascribed to the Mn^4+^/Mn^3+^ redox couple (Figure 4A). Peaks are much broader than those observed with the corresponding *bulk* LNMO electrodes. As discussed for the NMC532 case, the different shapes of the CVs could be explained by the thickness of the LNMO transistor channel material compared to the *bulk* electrode. In the range 3.5–4.3 V versus Li^+^/Li, the voltammetric currents are almost proportional to the V_gs_ scan rate, thus suggesting a pseudocapacitive behavior at such potentials.

Figures 4B–4D demonstrate the transfer characteristics of the LNMO IGTs at different V_gs_ scan rates. I_ds_ increases above V_gs_ = 0 V (3 V versus Li^+^/Li). I_ds_ reversibly increase with the de-lithiation (oxidation) of the LNMO channel, indicating the parallel increase of electronic conductivity of the LNMO composite with the lithiation.

The values of the accumulated charge during the forward scan in the transfer curves (obtained by the integration of *I*_*gs*_ versus time) were 3.7 × 10^−4^ C (5 mV s^−1^), 1 × 10^−4^ C (20 mV s^−1^) and 1.7 × 10^−5^ C (100 mV s^−1^). The carrier densities of the LNMO-based composite channel were calculated using Equation 1 and were 1.1×10^15^, 7.4×10^15^ and 2.5 × 10^16^ cm^−2^ at a scan rate of 100 mV s^−1^, 20 mV s^−1^ and 5 mV s^−1^. The values of the mobility for LNMO-based composite IGT channels at scan rates of 5, 20 and 100 mV s^−1^ were ca. 4.1 × 10^−3^, 1.5 × 10^−2^ and 8.8 × 10^−2^ cm^2^ V^−1^ s^−1^. The threshold voltages at the three different scan rates considered were located at approximately 3 V versus Li^+^/Li. The device ON/OFF ratios were ca. 1.1 for all the scan rates.

The LNMO-IGT output characteristics (Figure S7) were conducted for V_gs_ ranging between 0 and -1.4 V, corresponding to channel potentials ranging from 3 to 4.4 V versus Li^+^/Li. As for NMC532-IGTs, a linear response of I_ds_ with V_ds_ is observed, which indicates the ohmic nature of the LNMO-based composite channel material. The resistance of the channel (R_ds_), calculated by the slope of the I_ds_ curves as a function of V_ds_ (Table S2), indicates that the electronic conductance of the LNMO composite material reversibly increases with the de-lithiation degree.

In conclusion, our work demonstrates that it is possible to follow the change of the electronic transport of LIB electrodes in operando, i.e., during materials’ de-lithiation/lithiation, by using an ion-gated transistor (IGT) configuration. We demonstrated this approach by investigating LIB composite cathodes as the transistor channel materials with LP30 electrolyte, typically adopted in commercial LIBs and featuring Li^+^ as the unique cation species, as the gating medium. The IGT approach offers the opportunity to measure electronic transistor currents as disentangled from ionic ones, in LIB cathodes. The characterization of IGTs making use of LNMO- and NMC532-based composite transistor channel materials confirmed that, for these materials, the electronic conductivity increases with the decrease of the lithiation in the transistor channel (battery cathode) material. Furthermore, the characterization of NMC532- and LNMO-IGTs shows that the IGTs we studied work in depletion mode and the transistor (electronic) current can be reversibly modulated by fast lithiation/de-lithiation processes.

The IGT approach paves the way toward advanced in operando diagnostic tools, which are urgently needed to carefully follow the dependence of the electronic properties of battery electrodes on their state of charge. Such tools are expected to improve the sustainability of LIBs through the identification of optimized operation conditions, for optimized performance.

### Limitations of the study

This study on LIMs contributes to design advanced LIB diagnostic tools and Li-IGTs for neuromorphic computing. Chemical characterizations conducted in operando on LIMs in IGT configuration, e.g., XPS in operando, could provide insights on compositional changes during prolonged device operation.

## STAR★Methods

### Key resources table

**Table:** 

REAGENT or RESOURCE	SOURCE	IDENTIFIER
Chemicals, Peptides, and Recombinant Proteins		
NMC532	Gelon Lib.Co	CAS ID: 182,442-95-1
LNMO	NEI Corporation	CAS ID: 12,031-75-3
NMP	Fluka	CAS ID: 872-50-4
PVDF	Arkema	CAS ID: 872-50-4
Super C65	Imerys	CAS ID: 1333-86-4
LP30	Sigma Aldrich	ID PubChem: 329,765,784
Carbon Paper	Spectracorp	CAS ID: 7782-42-5
PICACTIF SUPERCAP BP10	Pica	CAS ID: 7440-44-0 | 102,186

### Resource availability

#### Lead contact

Further information and requests for resources and materials should be directed to and will be fulfilled by the lead contact, Francesca Soavi (francesca.soavi@unibo.it).

#### Materials availability

This study did not generate new unique materials.

### Method details

Identifiers of the reagents used in this work are given in key resources table.

#### Bulk NMC532 and LNMO electrodes preparation

Bulk NMC532 and LNMO electrodes were prepared using LiNi_0.5_Mn_0.3_Co_0.2_O_2_ (Shandong Gelon Lib.Co, China) and LiMn_1.5_Ni_0.5_O_4_ (NANOMYTE SP-10, NEI Corporation, USA) powders. According to the powders data sheets, the NMC532 powder featured a particle size of 9-12 μm and a nominal capacity of 150 mAh g^−1^ (between 2.5 and 4.5 V vs. Li^+^/Li). The particle size of LNMO was 4-7 μm and the nominal capacity was 125 mAh g^−1^ (between 3.5 and 5 V vs. Li^+^/Li).

Electrodes were fabricated using the polyvinylidene fluoride (PVdF, Kynar HSV900, Arkema) binder and processed with N-methyl Pyrrolidone (NMP, Fluka, >99.0%). The final composition of the composite electrode material was 80% NMC532 or LNMO, 10% conductive carbon (Super C65, Imerys), and 10% PVdF. The composite mass loading was 3-5 mg cm^−2^. LMOx and the conductive carbon powders were dry milled at 250 rpm for 5 min using a planetary ball miller (FRITSCH, Pulverisette), and tungsten jar (12 mL) and spheres (10 spheres, 5 mm diameter). Then, a solution of PVdF in NMP was added to the jar, resulting in a slurry that was milled at 250 rpm for 1 h (30 min reverse). The slurry was subsequently cast on aluminum foil, dried at 60 °C overnight in a thermostatic oven, pressed and dried again under dynamic vacuum at 120 °C to eliminate any solvent traces. Finally, the electrodes were transferred and stored in a dry Ar box (MBraun, H_2_O, and O_2_<1 ppm).^27^^,^^28^

The electrodes were cut into 10 mm disks and tested in 2-electrode Swagelok Teflon-made cells with AISI 316L connectors. For the cyclic voltammetry, we used 200 μL of 1M LiPF_6_ in 1:1 (*v*/*v*) ethylene carbonate (EC):dimethyl carbonate (DMC) (LP30, Sigma Aldrich, Ludwigshafen, Germany), commercial Celgard 2300 separator, and metallic lithium as quasi-reference and counter electrode. The electrochemical tests were performed in a thermostat at 30 °C with a BioLogic VSP multichannel potentiostat/galvanostat.

#### Pristine NMC532 and LNMO films characterization

X-ray diffractograms were obtained with a Bruker D8 diffractometer with wavelength (CuK_a_) of 1.54 Å. For SEM studies, we used a FEG JEOL 7600F microscope. Most of the images were taken at an accelerating voltage of 5 kV with a ET secondary electron detector. The chemical analyses were also done at 5 kV. Observations (Figures S1–S4) agreed with those reported in.^27^^,^^28^

#### IGT fabrication and assembly

IGT channel material were based on NMC532 or LNMO composite electrodes. Figure 2 shows the IGT structure used in this work, based on a SiO_2_/Si substrate patterned with drain and source contacts. These contacts were made of a 40-nm-thick Au layer on a 5-nm-thick Ti adhesion layer, with an interelectrode distance (L) of 10 μm and a width (W) of 4000 μm. A polydimethylsiloxane (PDMS) well was placed over the patterned substrate. A carbon paper electrode (0.5 cm^2^) coated with activated carbon (PICACTIF SUPERCAP BP10, 0.5 mg cm^−2^) acting as the gate electrode was placed inside the PDMS well.^34^^,^^35^^,^^36^ The carbon paper was fixed with copper tape. The gate electrode was immersed in the well filled with the LP30-based electrolyte. IGT assembly took place in a N_2_ glove box (O_2_ and H_2_O < 5 ppm).

The channel was drop-cast from the NMC532- or LNMO-based slurries. In addition to NMC532 or LNMO powders, the slurries included carbon additive (Super C65), and PVdF in 8:1:1 mass ratio, dispersed in NMP. After casting, the samples were vacuum dried at 80 °C overnight. The average thickness of the NMC532 and LNMO films was 6 ± 3 μm and 14 ± 2 μm, respectively. The gate electrode was made of carbon paper (CP, Spectracorp 2050) coated with an ink of activated carbon (PICACTIF SUPERCAP BP10, Pica) and PVdF binder in NMP. The coating was thermally treated at 80° C for several hours to remove the solvent and water traces. The resulting mass loading of the carbon coating was 0.5 mg cm^-2^.^33^^,^^34^^,^^35^

#### IGT characterization

During the electrochemical studies in IGT configuration, the gate electrode acted as a counter and a quasi-reference electrode, and the channel material was positioned between source and drain as the working electrode.^11^^,^^34^^,^^35^ The carbon gate potential was measured vs. lithium metal in the selected organic electrolyte and resulted in 3 V vs. Li^+^/Li. Hereafter, the channel potentials are given vs. Li^+^/Li. The characterization of the IGTs was carried out in a N_2_ glove box (H_2_O, O_2_<5 ppm) using a B1500A Agilent semiconductor parameter analyzer.
