# Supplementary material for: Electronic properties of lithium-ion battery cathodes studied in ion-gated transistor configuration
Source: iScience. 2022 Dec 27;26(1):105888. doi: 10.1016/j.isci.2022.105888 (PMC9860478; doi:10.1016/j.isci.2022.105888)
Supplement: Document S1. Figures S1–S7 and Tables S1 and S2 [file mmc1.pdf]

iScience, Volume *26*

Supplemental information

Electronic properties of lithium-ion battery cathodes studied in ion-gated transistor configuration

Federico Poli, José Ramón Herrera, Tian Lan, Prajwal Kumar, Clara Santato, and Francesca Soavi

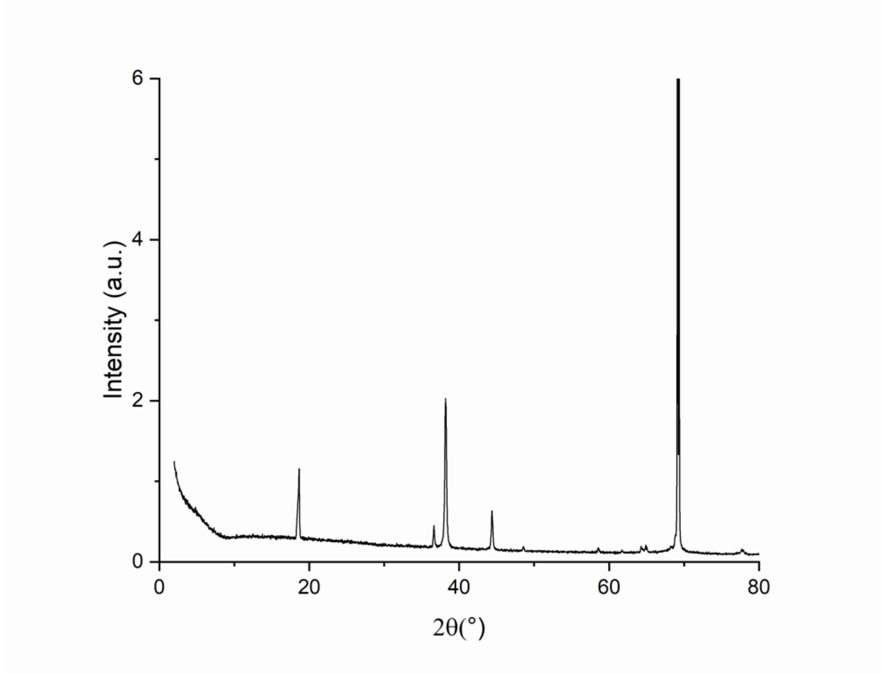

Figure S1. *XRD pattern of NMC532 composite deposited on SiO_2/Si. Related to Figure 2 and Figure 3.*

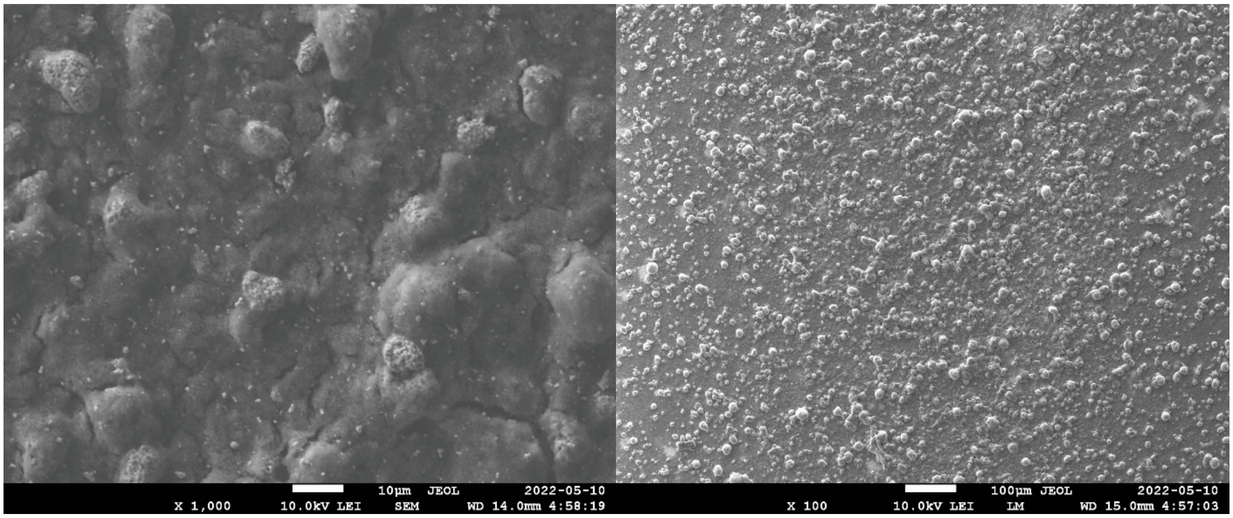

Figure S2. *SEM images of NMC532 composite deposited on SiO_2/Si substrate at different magnifications. Related to Figure 2 and Figure 3.*

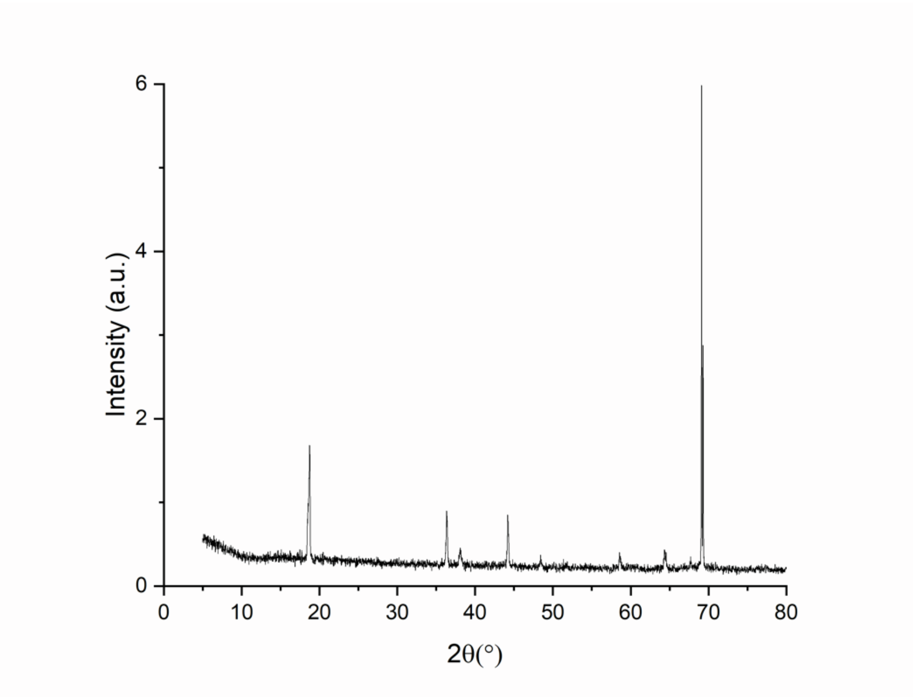

Figure S3. *XRD pattern of LNMO composite deposited on SiO_2/Si substrate. Related to Figure 2 and Figure 4.*

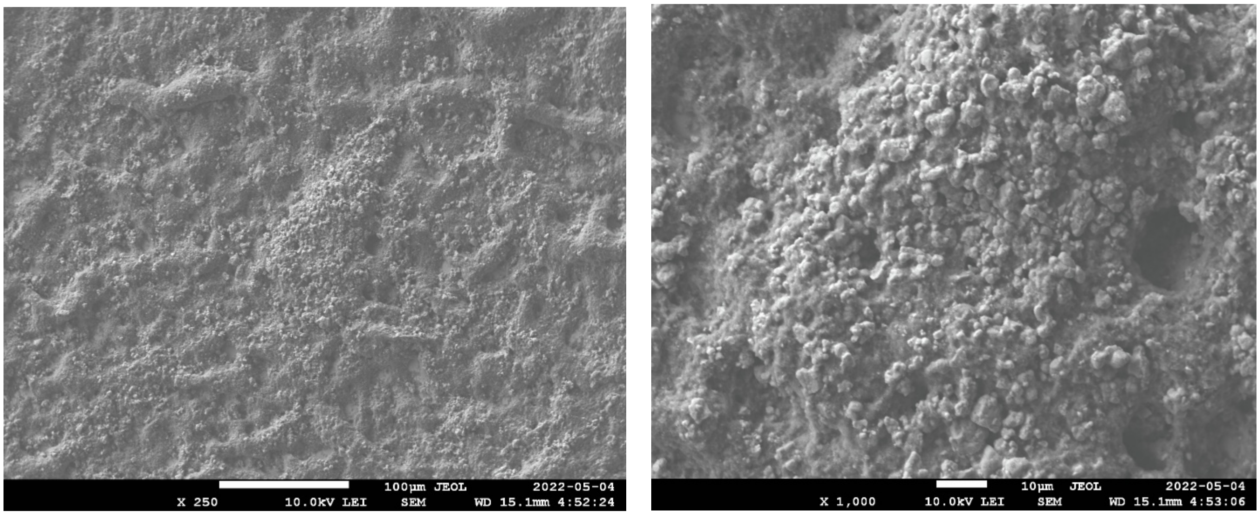

Figure S4. *SEM images of LNMO composite deposited on SiO_2/Si substrate at different magnifications. Related to Figure 2 and Figure 4.*

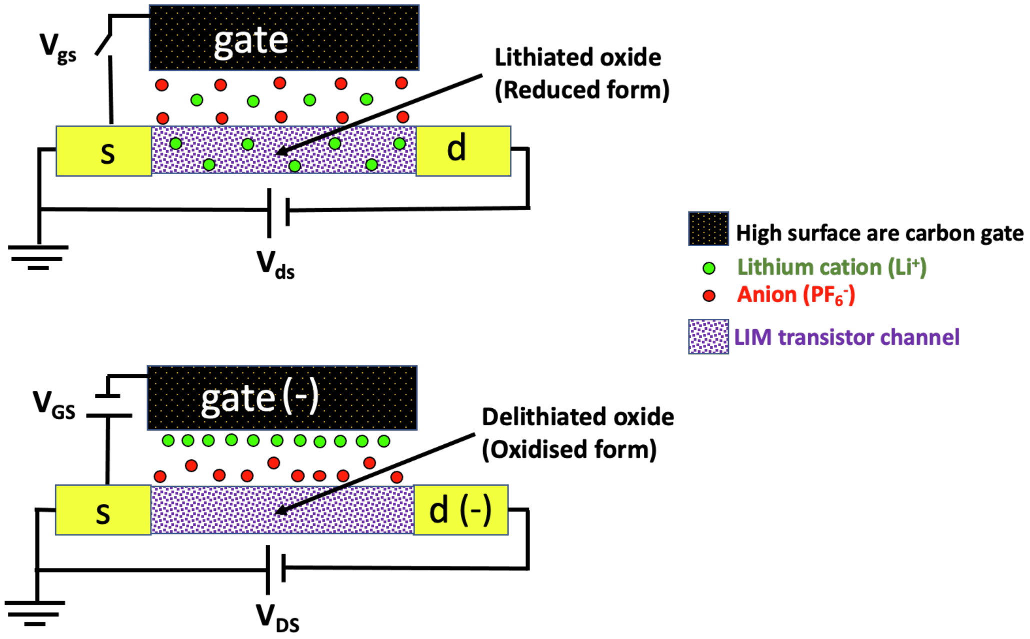

Figure S5. *Working principle of the IGT (top) without V_{gs} and (bottom) with V_{gs} bias applied. Before application of V_{gs}, the channel is lithiated (top). When a negative V_{gs} is applied, the channel is delithiated (bottom): Li^+ ions move towards the carbon gate, where an electrical double layer forms at the carbon/electrolyte interface. The value of I_{ds} depends on V_{gs} (i.e. on the state-of-charge of the LIM). Related to Figure 2.*

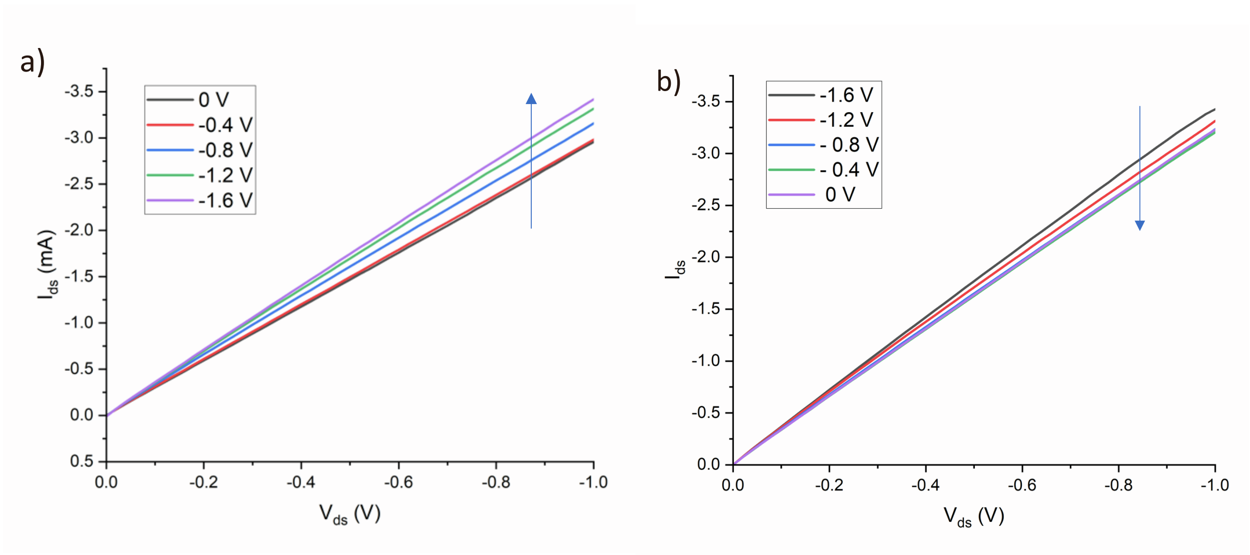

Figure S6. *Output curves of NMC532- based IGTs at V_{ds} scan rate of 20 mV s^{-1} for:* **a)** *forward scan with V_{gs} from 0 to -1.6 V, corresponding to potentials from 3 V to 4.6 V vs Li^+/Li;* **b)** *reverse scan with V_{gs} from -1.6 V to 0 V, corresponding to potentials from 4.6 V to 3 V vs Li^+/Li. Related to Figure 2 and Figure 3. Related to Figure 2 and Figure 3.*

Table S1. NMC532 *composite electronic resistance evaluated from the output tests at different V_{gs} during forward and backward V_{ds} sweeps at 20 mV s^{-1}, reported in Fig. S6. Related to Figure 2 and Figure 3.*

V_{gs} (V *vs.* Li/Li^+)	R (Ω) Forward	R (Ω) Backward
0.0 V (3.0 V *vs.* Li/Li^+)	340	310
-0.4 V (3.4 V *vs.* Li/Li^+)	336	312
-0.8 V (3.8 V *vs.* Li/Li^+)	318	310
-1.2 V (4.2 V *vs.* Li/Li^+)	302	303
-1.6 V (4.6 V *vs.* Li/Li^+)	292	290

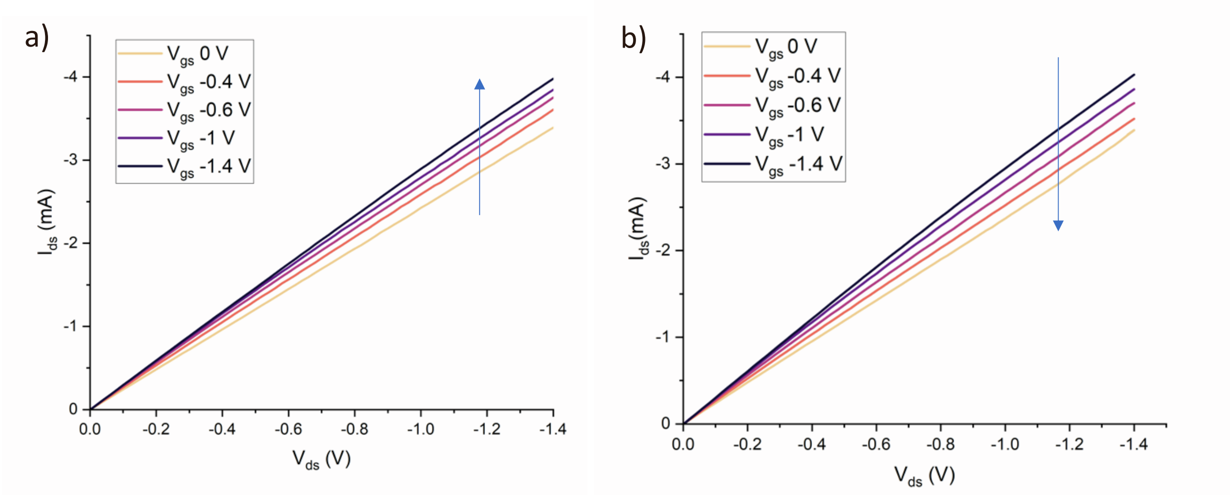

Figure S7. *Output curves of LNMO-based IGTs at V_{ds} scan rate of 20 mV s^{-1} for:* **a)** *forward scan with V_{gs} from 0 to -1.4 V, corresponding to potentials from 3 to 4.4 V vs Li^+/Li;* **b)** *reverse scan with V_{gs} from -1.4 to 0 V, corresponding to potentials from 4.4 to 3 V vs Li^+/Li. Related to Figure 2 and Figure 4.*

Table S2. LNMO *composite electronic resistance evaluated from the output tests at different V_{gs} during forward and backward V_{ds} sweeps at 20 mV s^{-1}, reported in Fig. S7. Related to Figure 2 and Figure 4.*

**V_{gs} (V *vs.* Li/Li^+)**	**R (Ω) Forward**	**R(Ω) Backward**
0.0 V (3.0 V *vs.* Li/Li^+)	412	417
-0.4 V (3.4 V *vs.* Li/Li^+)	389	400
-0.6 V (3.6 V *vs.* Li/Li^+)	375	380
-1.0 V (3 V *vs.* Li/Li^+)	365	363
-1.4 V (4.4 V *vs.* Li/Li^+)	351	346
